# Investigating the Association Between Adolescent Internet Addiction and Parental Attitudes

**DOI:** 10.3389/ijph.2022.1605065

**Published:** 2022-10-10

**Authors:** Mehmet Bilge, Gülten Uçan, Hakan Baydur

**Affiliations:** ^1^ Mehmet Akif Ersoy University, Burdur, Turkey; ^2^ Manisa Celal Bayar University, Manisa, Turkey

**Keywords:** adolescence, internet addiction, parental attitude, structural equation modeling, democratic attitude

## Abstract

**Objectives:** In this study, the association between parents’ attitudes and internet addiction among adolescent high school students was investigated through some sociodemographic variables.

**Methods:** The research was carried out with 385 students studying in four high schools in western Turkey. Sociodemographic characteristics, parental attitude scale and internet addiction scale were used in the study. Descriptive statistics, logistic regression analysis and structural equation modeling analysis were applied.

**Results:** The results of multivariate logistic regression analysis demonstrated that there was a significant relationship between the combined mean score obtained from the Parental Attitude Scale and the mean score obtained from the Internet Addiction Scale, and that authoritarian parenting attitude increases the risk of internet addiction [OR = 1.70 (95% CI: 1.33–2.18)]. In the structural equation modeling analysis, the model summary fit values were determined to be at a good level (χ^2^/df = 2.86, GFI = 0.97, RMSEA = 0.073) regarding the relationship between parental attitude and internet addiction.

**Conclusion:** Adolescents’ internet addiction levels changes related to their parents’ democratic attitude evolve.

## Introduction

New communication technologies that make life easier and become an integral part of life also bring about psychopathological problems such as internet addiction (IA), which includes all pathologic online activities in the Diagnostic and Statistical Manual of Mental Disorders (DSM) IV has been defined an “impulse control disorder not including intoxicating substances” [1]. This health problem, the product of human-technology interaction, is also called pathological [2, 3], problematic [4–6], excessive [7] compulsive internet use [8, 9]. In the section titled “Conditions for Further Study” in DSM-V, internet addiction (IA) is listed among behavioral addiction types under the heading “internet gaming disorder.” Among the diagnostic criteria of the IA are an individual’s inability to limit his or her desire to enter the internet, the continuous increase of the time spent on the internet (tolerance), the failure of efforts to limit the duration of internet use, taking refuge in the internet to get rid of the dysphoric mood such as anxiety, guilt, and desperation, exhibiting withdrawal symptoms when away from the internet, continuing to use the Internet despite its negative effects on work, education or private life, and trying to mislead his or her relatives about the duration of Internet use [1, 10–14].

Although internet addiction is a type of addiction observed in all age groups, it is accepted that children and adolescents are at the highest risk [15–17]. Internet is a convenient tool that adolescents, free from social concerns in the process of identity formation, can use for several purposes such as expressing themselves in the virtual environment, communicating and developing relationships with others, enhancing their social skills, having fun, and learning thanks to opportunities which enable them to hide their identity and to meet on platforms specific to common interests [18, 19]. Therefore, as the rate of internet use among adolescents is increasing all over the world so is the incidence of IA [20–22].

Adolescents are more vulnerable to the negative effects of the internet leading to addiction because of the ongoing cognitive, emotional, and social development processes [4, 20, 23, 24]. Internet-addicted adolescents have lower academic achievement than non-addicts [16, 25], and they may have attention deficit and hyperactivity disorder [26]. It was also observed that among internet-addicted adolescents, emotional and behavioral problems arose [27], that the risk of depression increased [28], and that they were more susceptible to negative emotions such as social phobia, anxiety, loneliness, social isolation, and suicidal ideation. In some other studies, it has been shown that adolescents addicted to the Internet display more aggressive attitudes [29, 30].

Relationships within the family and parents’ attitudes—in other words, the emotional climate—play a determining role in the psychosocial and physically healthy development of children [31, 32]. In the literature, parents’ child-rearing style—that is, attitude—is examined in the following four main categories “authoritative”, “authoritarian”, “permissive” and neglecting/rejecting attitudes [33–35]. These categories are defined by the relationship between the interest (acceptance) and control (discipline) [33, 36]*.* Parents with a democratic attitude display a high interest and a disciplined approach and try understand their children’s feelings. Children who are raised in a democratic style tend to have high self-confidence, self-esteem, sense of responsibility, independence, and academic success, and to establish positive friendships. Parents who adopt an authoritarian attitude do not show emotional interest and support to their children and expect their children to completely fulfill their orders. While the permissive parental attitude sounds like the best upbringing style, it often has negative consequences, because their children who grow up with warm care and unlimited understanding do not need to correct their wrong behaviors. Children of negligent parents who show inconsistent discipline and interest are dragged into crime and are prone to alcohol and substance abuse.

Research shows that there is a relationship between excessive internet use in adolescents and family characteristics, family communication, excessive parental protection and monitoring [37]. In particular, it has been reported in recent studies that there is a relationship between parental attitudes and internet addiction. In the study of Doğan et al., it was reported that protective-demanding and authoritarian parental attitudes increase internet addiction [38]. A similar result was found in Aksoy and Koçtürk’s study between protective-demand family type and internet addiction [39]. In another study, it is seen that adolescents with authoritarian and careless family type have higher internet addiction scores [40]. These findings show that family type has an important role in adolescent internet addiction. In addition to the existence of the relationship between the attitude of families towards adolescents and internet addiction, its severity should also be determined. In other words, the relationship between parental attitude and internet addiction needs to be handled and examined within the framework of a model.

In the present study, the relationship between internet addiction among adolescents and the attitude displayed by their parents while they raised their children and sociodemographic properties was investigated.

## Methods

In this cross-sectional study in which the relational screening model was used, the aim was to investigate the relationship between IA in adolescents, and parental attitudes and certain sociodemographic variables.

### Participants

The population of the study included students studying at high schools in the City Center of Manisa province in the 2018–2019 Academic Year. It was calculated according to the results of the research conducted by Doğan et al. in which the relationship between parental attitude and internet addiction was tested. By using group means and standard deviations in the calculation, the sample size consisted of 190 individuals at 95% power, 0.001 alpha error level. [38].

In order to achieve the specified sample size from the study population, the high schools in Manisa City Center were classified in terms of the socio-economic level of the residential areas in which they were located. In the second step, four different high schools, each representing a different socio-economic level, were selected using the random sampling method. The study data were collected from 354 students studying in the selected schools with the data collection tools used in the study.

### Variables of the Study

While the dependent variable of the study is IA, its independent variables are parental attitude and other sociodemographic characteristics.

### Measures

The study data were collected using the Personal Information Form developed by the researchers of the study, Parental Attitude Scale and Internet Addiction Scale.

### Parental Attitude Scale

The scale developed by Kuzgun [41], and revised by Kuzgun and Eldeleklioğlu [42] is used to assess attitudes displayed by the parents of high school and university students while they raise their children. The scale consists of 40 items and 3 sub-dimensions, namely “democratic”, “protective” and “authoritarian”. The responses given to the items are rated on a 5-point Likert type scale ranging from 1 (non-relevant) to 5 (totally relevant). The internal consistency coefficients of the “democratic”, “protective” and “authoritarian” sub-dimensions are 0.90, 0.75 and 0.72, respectively [42]. In our study, the internal consistency coefficients were found to be 0.93, 0.84 and 0.80, respectively. In this study, the principal components analysis was used in order to obtain a combined parental attitude score from the scores for the sub-dimensions of the PAS. While the decrease in the combined parental attitude score indicates the authoritarian parental attitude, the increase indicates the democratic parental attitude, and the score close to the mean score indicates the protective parental attitude.

### Addiction Profile Index Internet Addiction Form

The APIINT developed by Ögel et al. in 2015 to determine the level of Internet addiction, is a measurement tool that complies with many international addiction diagnosis criteria, and can be used for clinical purposes [43], It consists of 18 items questioning the individual’s evaluations regarding the last 3 months. The responses given to the items are rated on a 5-point Likert scale ranging from “1 = never” to “5 = almost always.” The higher the score obtained is the higher the IA level is. The subscales of the scale are “frequency of internet use”, “diagnostic criteria of addiction,” “the impact of internet use on life,” “craving for internet use” and “motivation to reduce internet use.” The Cronbach’s alpha coefficient is .88 for the reliability of APIINT, and ranges between 0.64 and 0.77 for the subscales. Item-total score correlation coefficients are between 0.44 and 0.68, and the test-retest correlation is .85 for the overall scale (*p* < 0.01). When the cut-off point determined for the APIINT is 2 points, the sensitivity value of the scale is 0.90, the selectivity value is 0.90, the positive predictive value is 0.99, and the negative predictive value is 0.61.

### Personal Information Form

The items of the form developed by the researchers question the participants’ socio-demographic characteristics such as sex, age, year at school, academic achievement, family income, education level of the parents, and the purpose, habit and frequency of their internet use.

### Data Analysis

Findings obtained from the study were presented as number and percentage distributions, arithmetic mean ± standard deviation, minimum and maximum values. Spearman correlation analysis was used to determine the relationship between the mean scores for the overall APIINT and its subscales and the mean scores for the overall PAS and its subscales. The logistic regression analysis was used to determine the relationship between the independent variables and the IA status obtained in the classification performed by taking into account the cut-off point for the overall APIINT score. Findings are presented with Odds Ratio and 95% Confidence Interval values. In the final analysis, whether the relationship between parental attitude and addiction profile fitted a model was tested by the structural equation modeling. In the results obtained in the structural equation modeling, the goodness of fit values of the model and the direct and indirect effects of the variables are presented. In the analysis, LISREL 9.1, Stata 14.0 and SPSS 25.0 for Windows were used.

## Results

### Sample

The mean age of the participants was 14.9 ± 0.755 years. Of the participants, 55.9% were girls. When the participants were asked to evaluate their academic achievement status, 51.1% described it as “very good,” 33.3% as “good” and 15.5% as “moderate or bad.” As for the participants’ parents’ education level, the mothers of 51.7% of the participants and the fathers of 43.8% of the participants had “primary school education or below.” Of the participating students, 87.0% had at least one sibling, and 74.9% had their own room at home.

As for the ownership status of communication tools, 92.9% of the participants had a smart phone, and 76.6% had a computer. When they were asked about their purpose of using the internet, they said that they used it to access social networking sites (67.3%), to watch videos (54.3%), to do homework (43.8%) and to play games (33.5%). Of the participants, 48.6% used the internet for 1–3 h a day, 22.3% for 4–5 h, 14.1% for 6 h or more, and 10.7% for less than an hour. The mean score obtained from the Development of the Addiction Profile Index Internet Addiction Form by 40.7% of the students was above the cut-off point of 2.

### Analytical Results

The results of the correlation analysis performed to compare the mean scores the participants obtained from the overall APIINT and PAS and their subscales, and the combined index scores is given in Table 1. As is seen in the table, the mean scores for the overall APIINT and its subscales were negatively correlated with the democratic parent attitude score, and positively correlated with the protective and authoritarian attitude scores. There was a low but significant correlation between the democratic parental attitude score and the APIINT subscale scores (−0.252). The highest correlation was between the democratic parental attitude score and the mean score for the impact of internet addiction on life subscale of the APIINT (0.401), and the correlation was at a moderate level. While the level of the correlation between the APIINT total score and the protective attitude score was 0.268, it was 0.328 between the APIINT total score and the authoritarian attitude score. The highest correlation coefficient is in the effect on life sub-dimension of APIINT. The level of the correlation between the APIINT total score, and the combined PAS score, the signs of addiction and the impact of internet use on life subscales was 0.316, 0.283, and 0.466, respectively (Table 1).

**TABLE 1:** Correlation between the internet addiction scale and the parental attitude (Manisa, Turkey. 2018).

	Democratic attitude	Protective attitude	Authoritarian attitude	Combined PAS
Frequency of internet use	−0.056	0.090	0.154**	0.109*
Diagnostic criteria of addiction	−0.250**	0.215**	0.301**	0.283**
Impact of internet use on life	−0.401**	0.371**	0.450**	0.466**
Craving for internet use	-0.139**	0.181**	0.215**	0.191**
Motivation to reduce internet use	−0.097	0.070	0.051	0.081
**APIINT**	−0.252**	0.268**	0.328**	0.316**

*p < 0.05; **p < 0.01. Values in bold are statistically significant results.

Those whose scores were above the cut-off point determined as 2 for the APIINT were considered as internet addicts, Results regarding to the comparison of internet addiction with the PAS score and sociodemographic variables performed through the univariate and multivariate analysis are given in Table 2.

**TABLE 2:** Relationship between internet addiction and combined parental attitude score and sociodemographic variables (Manisa, Turkey. 2018).

Variables	Crude OR (95% CI)	Adjusted OR (95% CI)
Combined PAS score (Numeric)	1.64 (1.31–2.05)**	1.70 (1.33–2.18)**
Sex (Men)	0.77 (0.50–1.18)	0.51 (0.31–0.83)**
Age (≥15 years)	1.43 (0.80–2.56)	1.47 (0.78–2.78)
Academic achievement (Very good)	ref	ref
Academic achievement (Good)	1.97 (1.22–3.16)**	1.76 (1.05–2.96)*
Academic achievement (Bad)	1.52 (0.82–2.82)	1.03 (0.51–2.09)
Mother’s education status (Primary school and below)	0.81 (0.53–1.24)	—
Father’s education status (Primary school and below)	0.62 (0.41–0.96)*	0.70 (0.43–1.13)
Mother-father (Separate)	1.62 (0.79–3.33)	—
Perception of the family’s economic situation (Poor)	1.29 (0.84–1.97)	—
Having a sibling (yes)	1.20 (0.63–2.27)	—
Having a private room at home (no)	1.19 (0.73–1.93)	—
Communication tools the participant has		
Smart mobile phone (yes)	1.50 (0.63–3.57)	—
Personal computer (yes)	0.98 (0.60–1.62)	—
Tablet (yes)	0.99 (0.64–1.51)	—
Game console (yes)	1.15 (0.65–2.06)	—
Control		
Does the family restrict his or her use of communication tools? (No)	0.98 (0.63–1.52)	—
Does the family control his or her internet use? (No)	1.20 (0.78–1.84)	—
Purpose of using the Internet		—
Access to social media (yes)	1.44 (0.91–2.29)	—
Playing games (yes)	1.17 (0.75–1.84)	—
Watching videos (yes)	1.29 (0.84–1.99)	—
Doing homework (yes)	0.63 (0.41–0.97)*	0.49 (0.31–0.80)**

*p < 0.05; **p < 0.01.

According to the results of the univariate analysis, the variables having a significant relationship with IA were as follows: good academic achievement [OR: 1.97 (1.22–3.16)], father’s having secondary education and above [0.62 (0.41–0.96)] and the use of the internet for doing homework [0.63 (0.41–0.97)], In the multivariate analysis, except for the father’s education, similar results were obtained, According to the results of the univariate analysis, the combined parental attitude score of the participants who were internet addicts was 1.64 (1.31–2.05) times higher than was that of the participants who were not internet addicts, In the multivariate analysis, this probability was 1.70 fold (1.33–2.18), In other words, as the combined parental attitude score shifts towards authoritarianism, so does the probability of the children’s being internet addicts, In this context, it was determined that parental attitude had a significant effect on the IA level of children, according to the results obtained both in the correlation analysis, and in the univariate and multivariate risk analysis.

**FIGURE 1:**
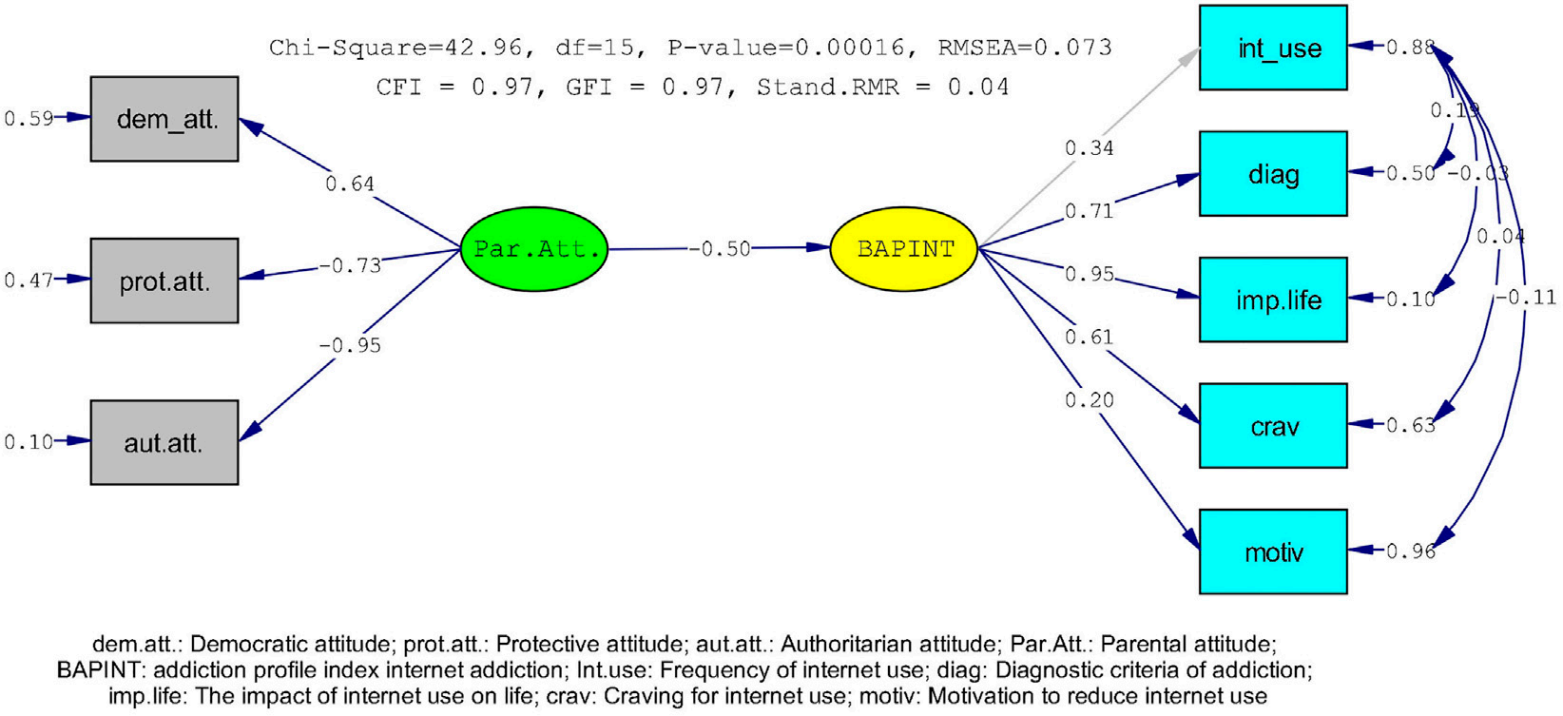
Results of the analysis of the relationship between parental attitude and internet addiction profile through structural equation modeling (Manisa, Turkey. 2018).

The results obtained from the analysis of the relationship between parental attitude and internet addiction profile through structural equation modeling are shown in Figure 1, The results of the summary of the goodness of fit indices revealed that the fit values of the model were χ^2^/df = 2.86, CFI = 0,97 and GFI = 0.97, The error criteria obtained from the model indicated good model fit (RMSEA = 0.073 and Stand, RMR = 0.047), It was determined that the conceptual model and the measurement model exhibited a harmonious structure, The effect of parental attitude, one of the variables in the model, on the Internet addiction profile was as follows: β = −0.50, The standardized total effect of parental attitude on the frequency of internet use, diagnostic criteria of addiction, the impact of internet use on life, craving for internet use and motivation to reduce internet use subscales of the IA profile were as follows: β = −0.17, *β* = 0.26, *β* = −0.33, *β* = −0.33 and *β* = −0.11 (*p* < 0.05) respectively, The results obtained demonstrated that the parental attitude had separate effects on the IA profile and its subscales.

## Discussion

In the present study, it was aimed to investigate whether there was a significant relationship between certain sociodemographic variables, and parents’ attitudes while they raise their children and adolescents’ IA levels. In line with this purpose, the variables examined in relation to the parental attitude and IA level were the participants’ sex, age, academic achievement, ownership of communication tools and internet access opportunities, In addition, the family’s income level, togetherness and education status of the parents were also analyzed, In the literature, of the studies conducted in different countries and with different samples, while some indicated that the IA level was higher in men [44, 45] some indicated that it was higher in women [27] however, in the present study, as in Dikmen and Tuncer’s [46] study, no significant correlation was determined between sex and IA, The differences between studies may have stemmed from the fact that cultural characteristics and some specific factors (family problems, depression, level of coping with stress, attention deficit and hyperactivity etc) affected IA level depending on sex. Therefore, as stated by Anderson et al, IA differs from one person to another not because of the sex variable but because of the interaction between push factors (escape from life and stressors, etc) and pull factors (low cost, ease of use, communication opportunities, etc.) [47].

Internet addiction was higher in the participants aged ≥15 years [crude OR 1.43 (95% CI: 0.8–2.56) times higher, adjusted OR 1.47 (95% CI: 0.78–2.78) times higher]. However, as in the study of Çakır et al, the relationship between age and IA was not significant. Contrary to this finding, in the literature, studies aimed at determining that the incidence of IA increases as the age increases have also been conducted [27]. This situation is explained by the fact that parental control decreases with advancing age in adolescents, that they use the internet as a medium where they can maintain increasing peer relationships, and that they wish to use the internet more as a means of relaxation due to their increased responsibilities and thus their stress load. That there is no relationship between age and the IA in the present study can be explained by the structure of the education system in Turkey which focuses on preparing students for exams and promoting their academic achievement during the last year of high school, in other words, as their age increases. As students pass their classes, they have to study more and reduce the time they spend for their social lives, of which the internet is a part, in order to get prepared for and pass the university entrance exam (Note: In Turkey, a student to enter a university should take the university entrance exam and pass it).

At the end of the study, no significant relationship was found between IA and the participants’ perceived income levels [OR = 1.29 (95% CI: 0.84–1.97)]. As Kısa stated [48] this finding can be associated with the low cost of internet use. According to the Turkish Statistical Institute (TurkStat) data, as of 2020, the rate of internet usage in the 16–74 age group has reached 79%, and the opportunity to access the internet at home has reached 90.7% [49]. Therefore, within this context, it can be assumed that the increase in adolescents’ opportunity to access the Internet in general may have led an increase in IA.

In the present study, there was no significant relationship between the IA levels of adolescents and their parents’ being together or separated [OR = 1.62 (95% CI:0.79–3.33)], which is compatible with the finding of Doğan’s study [50]. In other words, it can be said that parents’ togetherness has *no effect* on adolescents’ internet use. On the other hand, in Kısa’s study [48] IA was higher in students whose parents were separated than was that in students whose parents were together, which was probably due to the fact that one of the control mechanisms in the former students’ family was missing.

In the present study, IA was significantly related not to the mother’s but to the father’s education level. This is probably because as the father’s education level increased, so did the economic opportunities he provided for the family, which may have increased the opportunity to access and use of internet, and thus the level of IA. Similarly, in Aslanidou and Menexes’s study [51] the adolescents whose fathers had high school or higher education were able to access the internet more than were the adolescents in other groups.

The analysis of the IA levels of the participants in terms of the tools enabling them to access the internet demonstrated that the mobile phone owners obtained higher mean scores from the overall APIINT and its “diagnostic criteria of addiction” subscale.

The analysis of the IA levels of the participants in terms of the tools enabling them to access the internet demonstrated that the mobile phone owners obtained higher mean scores from the overall APIINT and its “diagnostic criteria of addiction” subscale. The comparison of the participants who had a computer, tablet or game console with those who did not have such tools demonstrated that there was a significant difference between their IA levels in terms of the mean scores they obtained from the “motivation to reduce internet use” subscale of the APIINT. In the literature, the findings obtained in studies investigating the relationship between the ownership of communication tools and the IA levels differed from one sample to another. For example, while in some studies, students who had a computer and opportunity to access the internet at home obtained high IA scores [45] in some other studies, no relationship was determined between the two variables [52]. Based on the findings of the present study, it is possible to assert that IA levels are higher in adolescents who have more communication tools due to the time they spend on the internet. Internet, which is an indispensable part of adolescents’ daily life, carries the risk of creating addiction depending on the purpose and duration of internet use. In the present study, using the internet to access social networking sites, to watch videos and to play games was determined to increase the participants’ IA risk but did not have a significant effect. However, it was observed that the IA levels of the participants who stated that they used the internet to do homework were significantly lowe [OR = 0.63 (95% CI:0.41–0.97)]. In Yüksel and Yılmaz’s [53] and Canoğulları and Güçray’s studies [19] conducted with students to determine their IA levels, IA levels of the participants who used the internet to play games and chat in the former study, and IA levels of the participants who used the internet to play games and to access social networking sites in the latter study were higher than IA levels of the participants who used the internet to do homework or research.

The analysis of the findings revealed that while internet addiction levels decreased among the adolescents whose parents displayed a democratic attitude, IA levels increased among the adolescents whose parents displayed authoritarian or protective attitudes. In other words, adolescents who perceive their parents as authoritarian or overprotective are more likely to have IA than those who feel they have a democratic relationship with their parents. In the structural equation modeling in which the univariate and multivariate analysis were performed, the combined parental attitude score also supports this finding. In the literature, studies emphasizing the similar relationship between parental attitude and the IA levels of children of different age groups have been conducted [54, 55]. On the other hand, in Canoğulları and Güçray’s study [56] IA levels of the adolescents who described their parents as ‘controlling parents’ were higher than were those of the other adolescents. The reason for this can also be considered as the parents of adolescents who have the ability to limit their internet use do not need to display an authoritarian or protective attitude. In other words, adolescents who are afraid of being punished, condemned, or limited by their parents are more likely to be internet addicted. Conversely in Kısa’s study [57] addiction levels of the students whose Internet use was restricted were lower than were those of the students whose internet use was not restricted. The findings can be interpreted as that even though the increase in the risk is insignificant in adolescents whose internet use is not controlled by parents, they may face serious problems progressing to addiction. In addition, the communication of the parents with the adolescents plays a protective role against IA [58].

Büyükşahin and Çelikkaleli’s study [59] the internet addiction level was higher in the children of “indifferent” parents than was that in the children whose parents displayed “democratic” or “authoritarian” attitudes. This may be due to the relatively low number of parents in the “indifferent” group. The need to be independent and autonomous from parents increases during adolescence [60]. However, not controlling the internet use of adolescents is also problematic, as is trying to be strictly controlled. Therefore, communicative, democratic parental attitude has a positive effect on children’s self-regulation and reduces IA [61]. Therefore, it can be said that a democratic control, which does not approach an authoritarian attitude, protects adolescents from IA.

Nevertheless, contrary to these findings, in their study Soh et al. determined no relationship between the parental attitude and the IA level of the adolescent, and they stated that this might be related to the adolescent’s effort to become an independent individual. According to the statistical comparisons made in this study, no relationship was determined between IA levels of adolescents and the restriction or control of the use of communication tools by the parents. The reason for this may be the predictive effect of adolescents’ personality traits related to autonomy, such as self-control and self-efficacy. The ability of adolescents to limit their internet use may have an effect on reducing IA regardless of their parents’ attitudes. A second explanation is that peers, which are known to play a vital role in adolescent development and behavior [60], are more likely to be more influential than the parent’s democratic or authoritarian attitude. Because, according to the study of Young et al. [62] low peer support is problematic in terms of its relationship with IA, as well as high parental support.

To sum up, IA in adolescents is a problem that is difficult to explain with parental attitude alone, but also needs to be examined in terms of biopsychosocial dimensions such as personal autonomy and relationships with peers.

### Limitations and Strengths

The main limitation of this research is that the evaluation of academic achievement is based on self-report and away from objectivity. It can be added that the responses to the questionnaires include subjective evaluations. In addition, although the sample size was calculated at the beginning, the results may not adequately reflect the reality due to sample selection. The strengths of the research include the initial calculation of sample size; analysis of the relationship between parental attitude and internet addiction with multivariate analysis. In addition, the results obtained with the “structural equation modeling,” which is a confirmatory approach, were reanalyzed.

### Recommendations to Policy Makers

Adolescents should be educated on correct internet use.
Parents should be educated on adolescence and adolescent behavior.
Educational programs should be organized to strengthen parent-adolescent relations.
The biopsychosocial dimensions of the problem should be studied in order to determine the variables that affect IA in adolescents.

### Conclusion

Excessive internet use is a behavioral addiction which not only threatens the physical and psychological health of individuals but also has negative effects on their social life. Although children have placed the relationships they have established with their peers at the center of their lives, they still need the support and attention of their parents during adolescence. The findings of the present study suggest that parents’ attitude is an important variable affecting adolescents’ IA levels. In the current study, it was determined that while the democratic parental attitude protected adolescents from IA and reduced their IA, protective and authoritarian parental attitude increased the risk.

Internet is an important and useful tool that plays a great role in the academic and social life of adolescents. Therefore, it is more beneficial to decide together with the adolescent how and how much the adolescent should use the internet, and to teach and encourage him or her to use the internet consciously. It is recommended that social workers should plan protective and preventive social work interventions that will increase the media literacy levels of adolescents and their families.
